# Surface Microbiology of Smartphone Screen Protectors Among Healthcare Professionals

**DOI:** 10.7759/cureus.1989

**Published:** 2017-12-26

**Authors:** Ibrahim Raza, Awais Raza, Syed Ahmad Razaa, Ahmad Bani Sadar, Ahmad Uzair Qureshi, Usama Talib, Gerald Chi

**Affiliations:** 1 Department of Medicine, King Edward Medical University Lahore, Pakistan; 2 Cardiology Department, Beth Israel Deaconess Medical Center; 3 Department of Medicine, Beth Israel Deaconess Medical Center

**Keywords:** microbiology, microbiology report, infection, commensal bacteria, pathogens, keywords: problematic mobile phone use

## Abstract

Background: The use of smartphones with touch screens has become a norm for healthcare professionals (HCP). The risk of smart screen contamination has been proven, and guidelines are available to deal with possible contamination. A large number of smartphone users apply plastic or glass screen protectors onto their mobile phone screens to prevent scratches. However, these materials are not scratch proof, and their antipathogenic properties have not been studied.

Methods: We have conducted a study to determine the frequency of smartphone screen protector contamination and compared the data with contamination on the bare area on the same mobile screens. The sample size included only HCPs working in acute care settings and having at least eight hours of exposure time every day.

Results: A total of 64 samples were collected, which reported 62.5% (n = 40/64) positive culture swabs from the protected areas of the screen and 45.3% (n = 29/64) from the unprotected area of the screen. Micrococcus and Gram-negative rods grew only on samples taken from the protected area whereas the bare area showed no such growth. There was no statistically significant difference in the frequency based on smart screen size, duration of use during duty hours, or the setting where it was used.

Conclusions: Smartphone screen protectors from healthcare providers may harbor pathogenic bacteria, especially in acute care settings. Coagulase-negative Staphylococci followed by Bacillus species were the most commonly yielded bacteria among house officers and postgraduate trainees in the present study.

## Introduction

With the introduction of the touchscreen smartphones genre, there has been an exponential growth in this sector. Smartphones are now considered to be an indispensable accessory in the social and professional life of healthcare providers (HCPs) [1-2]. According to United Nations Public Administration Network (UNPAN), approximately 30% of the world's population had a smartphone at the end of 2014. The number of smartphone users in the world exceeded the two-billion mark for the first time in 2014 [3]. Even more so, the World Health Organization (WHO) estimated 6.9 billion subscriptions globally in 2014 [4]. Consequently, HCPs have also adapted to the trend [2]. To address this situation, health policies have been derived regarding its use in clinical settings, ranging from strict prohibition hospital-wide to relatively lax guidelines [5-7]. The integration of smartphones into the healthcare settings has its implications in two major areas; electrical interference with equipment [7] and as a reservoir of nosocomial organisms particularly methicillin-resistant Staphylococcus aureus (MRSA). According to WHO, the pooled healthcare-associated infection (HCAI) prevalence in high-income countries is 7.6% while it is 10.1% (15.5%. if only high-quality papers are included) in low- and middle-income countries [8]. The device-associated infection rates for adult and pediatric ICUs are also several times higher in developing countries than National Healthcare Safety Network's rates [9]. Centers for Disease Control and Prevention (CDC), Association for Professionals in Infection Control and Epidemiology (APIC), The Writing Group of the Expert Panel of Canadian Infectious Disease, and WHO recommend hand hygiene as the “standard precaution” for the transmission of infectious agents [10-12] and the “primary measure” for prevention of HCAIs. However, the compliance with hand hygiene in HCPs is poor, reported with mean baseline rates ranging from 5% to 89% and an overall average of 38.7% [9].

Recent research shows the presence of microbial organisms on the surface of mobile screens of HCPs, with rates ranging from 60% to 95% [8,13-15]. Specific bacteria have been cultured and a wide spectrum of bacteria, including MRSA, was also cultured [13]. Hence, approaches to counter HCAI should not only include encouraging hand hygiene but also cater to the eradication of all the factors that might play a role as a reservoir of infectious agents. In one study, even after disinfecting the surface with 70% isopropyl alcohol, 8% showed positive cultures and 75% showed microbial growth after a week [16]. Attempts have been made to determine various factors to identify the potential risk posed by mobile phones. However, not much has been studied on mobile screen protectors as a risk factor for being a potential source of bacteria. This study was designed to determine the risk of harvesting pathogens from mobile phones and to compare their frequencies with or without the use of screen protectors.

## Materials and methods

Study design

A cross-sectional observational study was designed from June to December 2015 at Mayo Hospital, Lahore, Pakistan. Healthcare providers (HCPs) in acute patient care settings were included in the study. Acute care settings were defined as intensive care units (ICU), high dependency units (HDU), and the emergency department (ER). HCPs possessing a personal smartphone on a working shift of at least eight hours a day with access to Wi-Fi and/or cellular mobile data access were included. Only doctors using a screen protector for more than three months were included in the study. Screen protectors that had been changed less than three months ago were excluded to reduce selection bias. Those doctors who owned a smartphone less than three months old or using a non-touch-screen phone or working in an environment where mobile phones cannot be used, such as operation theaters, were also excluded. The purposive sampling technique was used. A total of 64 HCPs were enrolled in the study. The Institutional Review Board of King Edward Medical University, Lahore, approved the study. Written informed consent was obtained from the study participants. Confidentiality of data was maintained by demographic information collected by different team members and by making sure that the identity of the HCPs was kept confidential at the time of data analysis.

Microbiology laboratory analysis

After obtaining written consent and satisfaction of inclusion and exclusion criteria, swab samples were taken, one each from the screen protector area and the bare area of the same mobile phone. The samples were obtained in an S-shaped pattern, as specified by Centers for Disease Control and Prevention (CDC) in the guidelines for surface swab sampling. The collected sample was submitted for culture within 24 hrs in a culture lab with an ISO9001 certificate.

For the cultures, all standard procedures and precautions, as per Clinical and Laboratory Standards Institute (CLSI) 2015, were applied. An application was installed in the participant’s mobile phones, after proper informed consent, which calculated the total time of usage. Those who did not wish to install the application were asked to self-report their time period of mobile usage. In accordance with the CDC swab sampling protocol, two swab samplings were performed at the end of the shift of each HCP to ensure an adequate duration of exposure. The swab was placed in the Amies transport medium in a test tube and transferred to the laboratory within 24 hours. Mobile phone swabs from the bare and protected area were streaked onto two plates (blood agar and MacConkey agar mediums). Plates were incubated aerobically at 37°C for 48 hours. Isolated organisms were identified using Gram staining, morphology, catalase, oxidase, and all isolates were allocated to the appropriate genera. For the identification of Gram-negative bacteria, API 20E and API 20NE system (bioMérieux, France) was used. A slide coagulase differentiated Staphylococcus aureus from coagulase-negative Staphylococci. All lab tests followed protocols as established by the CLSI. The pathologist was blinded to the origin of the swab. The samples were sent with a unique identifier for later correlation by the authors at the time of data analyses.

Statistical analysis

The data were analyzed using SPSS statistical software version 21.0 (SPSS Inc., Chicago, IL, USA). Descriptive analyses were done for demographic data. Means were calculated for quantitative data with range and standard deviation. Frequencies for the type of screen protectors and how often these are changed have also been determined. A descriptive analysis of microorganisms cultured from either the protected or the unprotected area was performed as well.

## Results

A total of 64 participants (males: 38; females: 26) using a touchscreen mobile for at least three months were enrolled in the study. A descriptive analysis of the baseline demographics is provided in Table 1. House officers and postgraduate trainees from four major specialties were included with a frequency of 45% (n = 29/64) and 50% (n = 32/64) participants, respectively. Commonly used were tempered glass and plastic (50% versus 48.5%). Samples were collected from facilities where acutely ill patients presented more commonly with the majority collected from the emergency department followed by intensive care units, high dependency units, operation theaters, and wards taking acute patients (Table 1). No statistically significant difference was found in the size of the mobile used or the screen size in groups with positive growth (Table 2).

**Table 1:** Baseline characteristics Abbreviations: ER, emergency room; HDU, high dependency unit; ICU, intensive care unit; OR, operation room

Study Participants (n = 64)		
Sex	Males	38 (58.5%)
	Females	26 (41.5%)
Age	Mean ± standard deviation	27.17 ± 4.29 years
	Range	23 to 46 years
Specialty	Internal medicine	20/64 (30.8%)
	Surgery	32/64 (49.2%)
	Anesthesia	9/64 (13.8%)
	Cardiology	3/64 (4.7%)
Designation	House officers	29/64 (45%)
	Postgraduate trainees	32/64 (50%)
	Others	3/64 (5%)
Place of Duty	Emergency room (ER)	46/64 (71%)
	Operation room (OR)	7/64 (10.9%)
	Intensive care unit (ICU)	4/64 (6.3%)
	High dependency unit (HDU)	4/64 (6.3%)
	Ward duty (critical care beds)	3/64 (4.7%)
Frequency of positive growth	Protected area (growth vs. no growth)	40 vs. 24
	Bare area (growth vs. no growth)	29 vs. 35

**Table 2:** Frequency of bacteria on protected and bare areas

No.	Growth	Protected area	Bare area	P-value
1	No growth	24/64	35/64	0.08
2	Growth seen	40/64	29/64	0.45

A total of 62.5% (n = 40/64) of culture swabs taken from the screen protector area (protected area) resulted in some type of bacterial growth on culture plates as compared to 45.3% (n = 29/64) from the unprotected area (mobile screen directly) near the function keys. Micrococcus and Gram-negative rods grew only on samples taken from the protected area (i.e. glass or plastic protectors), whereas the bare area showed no such growth. The rest of the bacterial strains showing growth in both the areas, in different proportions, is summarized in Table 3. Different types of screen protector material were also compared. The frequency of positive culture for both were 61.3% positive cultures for glass as compared to 63.3% for plastic protectors. Out of five different types of bacteria found in total 40 subjects in positive cultures among protected surfaces (total subjects 64, no growth cases 24), coagulase-negative Staphylococci were found in 70% of positive cultures (28 out of 40). Similarly, out of three different types of bacteria found in total 29 subjects showing positive cultures among bare surfaces (n = 35/64), coagulase-negative Staphylococci were found in 58.6% (17 out of 29) of these cases, again making it the most frequently found bacteria among positive cultures in two main tertiary care hospitals of Lahore.

**Table 3:** Frequency percentages of commonly cultured organisms

Microbiology results	Protected surfaces (n = 40)	Culture results from bare area (n = 29)
Bacillus	20 (50.0%)	15 (51.7%)
Coagulase-negative Staphylococci	28 (70.0%)	17 (58.6%)
Diphtheroid	2 (5.0%)	3 (10.3%)
Micrococcus	1 (2.5%)	0 (0.0%)
Gram-negative rods	1 (2.5%)	0 (0.0%)

The contamination is maximum in case of HDU, which is 75% positive cultures from the protected area of mobile phone screens as compared to 65.2% in the emergency department, 42.9% in the operation theater, and 25% in the ICU. Whereas in cases of bare surfaces, infectivity chance is relatively low, the maximum chance of infectivity is in the wards i.e. 66.7%, which is followed by the emergency department and the operation theater with percentages of infectivity chances of 47.8% and 42.9%, respectively. Out of four specializations (medicine, surgery, anesthesia, and cardiology) in the acute care settings of tertiary care hospitals, the protected screens of the mobiles of subjects working in the anesthesia department were found to harbor more bacteria than in any other specialty, with a percentage of 77.8%. This was followed by cardiology and surgery, with percentages of 66.7% and 62.5%, respectively. This might be due to the fact that anesthesiologists have to work and manage patients in all types of acute care settings in tertiary care hospitals, be it surgery, medicine, emergency, operation theater, ICU, or HDU.

Methods used by the healthcare providers to disinfect their mobile phones were determined by the questionnaire method. Out of 64 subjects, 11 disinfected their mobile phones with alcohol sterile swabs. Out of these 11 subjects, four subjects disinfected one month before enrollment and showed no growth on either protected or bare area. The remaining seven subjects disinfected their mobile phones two to three months earlier and showed commensal organisms such as Bacillus and coagulase-negative Staphylococci (Table 4).

**Table 4:** Practices of healthcare providers to disinfect their mobiles

Disinfection	Frequency	Bacterial growth
Total disinfections	11/64 (17.1%)	–
1-month disinfection	4/64 (6.3%)	No growth
> 1-month disinfection	7/64 (10.9%)	Limited growth (mainly commensals e.g. bacillus)
Not disinfected	53/64 (82.9%)	Major chunk of bacterial growth seen (including coagulase-negative Staphylococci, Micrococcus, diphtheroids, Gram-negative rods)

The frequency of each bacterium found in our study corresponding to four different designations of healthcare personnel showed higher prevalence among HCPs directly in contact with patients. As the results suggest, 46 culture swabs out of 128 culture swabs (two swabs per person from 64 subjects) showed a growth of the coagulase-negative Staphylococci, out of which 47.8% (n = 22) positive cultures were from house officers and the same frequency i.e. 47.8% (n = 22) of positive cultures were found from postgraduate trainees. The result shows the frequency of positive cultures from the protected area with a screen protector is approximately double the bare area (Table 5).

**Table 5:** Frequency of different bacteria in different grades Abbreviations: HO, house officer; PG, postgraduate trainee

Protected area						
No.	Bacteria	HO	PG	Consultant	Nurse	Total
1	Bacillus	7	12	1		20
2	Coagulase-negative Staphylococci	15	12		1	28
3	Diphtheroids	2				2
4	Micrococcus	1				1
5	Gram-negative rods		1			1
Bare area						
No.	Bacteria	HO	PG	Consultant	Nurse	Total
1	Bacillus	6	8	1		15
2	Coagulase-negative Staphylococci	7	10	1		18
3	Diphtheroids	2	1			3
4	Micrococcus					
5	Gram-negative rods					
Total						
No.	Bacteria	HO	PG	Consultant	Nurse	Total
1	Bacillus	13 (37.1%)	20 (57.1%)	2 (5.7%)		35
2	Coagulase-negative Staphylococci	22 (47.8%)	22 (47.8%)	1 (2.1%)	1 (2.1%)	46
3	Diphtheroids	4 (80%)	1 (20%)			5
4	Micrococcus	1 (100%)				1
5	Gram-negative rods		1 (100%)			1

## Discussion

Nosocomial infections pose a serious threat to the health of patients in hospitals [17]. Increased virulence and resistance in pathogens warrants every possible measure to be taken to prevent the spread of infection. Objects in the use of healthcare providers (HCPs), such as stethoscopes and ballpoint pens, have been demonstrated to be possible sources of nosocomial infection [18-19].

Mobile phones have become so indispensable that now they are an integral part of our everyday life [1]. Initially, strict restrictions were laid down to curb mobile phone usage in hospital settings, especially in operation theaters, but these were relaxed later [5-7] because mobile phones have become an integral part our daily lives and help improve healthcare delivery [1-2]. Many studies have been conducted to determine the role of mobile phones, particularly their screens, in harboring and spreading pathogens [20-23]. Almost all of these studies have made no distinction between a bare screen and a mobile screen covered with a screen protector, which, actually, are two different substances with different physical properties.

Our cross-sectional study showed that the contamination rate of protected areas is more than the contamination rate of bare areas. However, the overall contamination rate was found to be 75 percent. Similar overall contamination rates were found by numerous studies (i.e. 70% to 78.6%) [15,23-25]. Higher contamination rates of 100% were reported by Selim et al. [21], Tagoe et al. [1], Ustun et al. [22], Pal et al. [26], and Kokate et al. [27]. In contrast, Mark et al. [14] reported a lower contamination rate of 60%.

The most commonly harvested bacteria were coagulase-negative Staphylococci, a finding consistent with prior studies of similar nature [14-15,24,27-28]. No MRSA was yielded, which was consistent with the results of previous studies [20,24,27]. Tagoe et al. [1] found the contamination rate of MRSA to be just 4%. While Venkatesan et al. [29] reported a lower MRSA rate of 2.14%, a higher rate of MRSA at 53.2% has also been described [11].

Staphylococcus epidermidis and Staphylococcus saprophyticus, although commensal and nonpathogenic, can pose pathogenicity if it gets a chance to enter the bloodstream through intravenous drug administration, dialysis, catheter insertions, or spinal anesthesia, which is a usual practice under acute settings in tertiary care hospitals across the developing countries. Staphylococcus epidermidis is a major cause of hospital-acquired infection. In cases of prosthetic heart valves or prosthetic joints, Staphylococcus epidermidis can cause endocarditis or osteomyelitis, respectively. Staphylococcus epidermidis is also an important cause of sepsis in neonates and peritonitis in patients undergoing peritoneal dialysis through an indwelling catheter. In fact, Staphylococcus epidermidis is reported to be one of the most common causes of cerebrospinal fluid shunt infection [30].

HDU had the highest rate of contamination, i.e. 75%, for protected areas. Heyba et al. [15] found a similar contamination rate (i.e. 73.7%) for HCPs working in ICUs, neonatal ICUs, and pediatric ICUs. One possible explanation might be that HDU receives critically ill patients that have a higher possibility of carrying more resilient pathogens compared to patients admitted with community-acquired infections.

Out of four specializations working in acute care settings in tertiary care hospitals, the protected screens of the mobiles of subjects working in anesthesia were found to harbor more bacteria than other specialties. One possible explanation is that HCPs working as anesthesiologists were exposed to a wider variety of patients compared with other specialties because of diverse interdepartmental rotation.

Bacterial growth was seen on the mobile phones of the subjects who had not disinfected their mobile phones at all. Subjects that had disinfected their mobile phones almost one month ago did not show any bacterial growth on either the protected or the bare surfaces of their mobile phones while those who disinfected their mobile phones more than one month ago showed commensal bacterial growth. The fact that mobile phones are personal and carried by each HCP adds to the potential of these devices to spread disease. In an interesting study, Smith et al. [18] demonstrated that the stethoscope that was designated to a room was statistically less frequently contaminated (45%) as compared to the HCP’s personal stethoscope. It was postulated that the one designated to the room underwent similar sterilizing procedures as other instruments in the room while the personal stethoscope rarely got cleaned or sterilized. This explains why the risk of contamination associated with personal objects, which, in this case, is a mobile phone could be much higher than shared objects. Findings from the present study suggest that disinfecting mobile phones at least once a month may be effective in restricting the growth of microorganisms and even commensals such as coagulase-negative Staphylococci.

In our tertiary care hospitals, house officers and postgraduate trainees were the ones exposed to the greatest number of patients on a daily basis in the acute care setting, so they were more prone to catching and then transmitting infection if they didn't take the necessary hygienic precautionary measures, such as using sterile gloves on patient encounter, washing hands properly after patient encounter, and sterilizing mobile phones at least once a month.

We cannot completely remove mobile phones from hospitals since they are not only a means of rapid communication but also add to overall health care provision to patients [13,20]. Future research efforts should focus on developing comprehensive guidelines regarding routine decontamination of mobile phones with alcohol or other effective means. HCPs should be educated about the possible risks associated with mobile phone usage in the hospital setting and should be encouraged to minimize them as much as possible. Manufacturers should provide clear recommendations for the decontamination of mobile phones. The use of sterile gloves should be encouraged and proper hand hygiene should be reinforced.

The study is limited by its use of a tertiary care hospital population and relatively small sample size. Information about the number of patients examined by each HCP, a factor that may confound the culture results, was not available. Sampling matching was achieved by using both the bare and protected screens of the same phone of a single subject simultaneously. To minimize the Hawthorne effect, different clinical settings of hospitals were visited randomly, unannounced, at different working hours. Future studies with a larger sample size should be performed in order to confirm these findings. Recently, several “antibacterial” and “antimicrobial” screen protectors have been developed that have a metallic (silver nanotechnology) component added to them. Further research should investigate the effectiveness of the antibacterial or antimicrobial screen protectors. However, in lower- or middle-income countries, such as Pakistan, the availability of such screen protectors is very limited.

## Conclusions

Smartphone screen protectors from healthcare providers may harbor pathogenic bacteria, especially in acute care settings. Coagulase-negative Staphylococci followed by Bacillus species were the most commonly yielded bacteria among house officers and postgraduate trainees in the present study.
